# Experimental analysis of mating patterns in a clonal plant reveals contrasting modes of self‐pollination

**DOI:** 10.1002/ece3.1801

**Published:** 2015-11-02

**Authors:** Yi Hu, Spencer C. H. Barrett, Da‐Yong Zhang, Wan‐Jin Liao

**Affiliations:** ^1^ State Key Laboratory of Earth Surface Processes and Resource Ecology and MOE Key Laboratory for Biodiversity Science and Ecological Engineering College of Life Sciences Beijing Normal University Beijing 100875 China; ^2^ Department of Ecology and Evolutionary Biology University of Toronto 25 Willcocks Street Toronto Ontario M5S 3B2 Canada

**Keywords:** *Aconitum*, bumblebee foraging, clonal growth, geitonogamy, mating patterns, modes of selfing

## Abstract

Hermaphrodite plants commonly practice self‐fertilization (selfing), but the mechanisms responsible vary depending on the mode of self‐pollination, pollinator behavior, and degree of clonality. Whether selfing occurs within (autogamy) or between flowers (geitonogamy) is of evolutionary significance because their fitness consequences differ. We used floral manipulations and genetic markers to determine the relative contribution of autogamy and within‐ versus between‐ramet geitonogamy to the selfing rate of the bumblebee‐pollinated, clonal herb *Aconitum kusnezoffii*. Data on flowering phenology and bumblebee foraging were also collected to determine opportunities for different modes of self‐pollination. Autogamy accounted for only 12% of the selfing rate with the remainder resulting from geitonogamy. Whole‐ramet emasculation of clones with multiple ramets reduced selfing by 78%, indicating that within‐ramet geitonogamy contributed significantly (68%) to total selfing. Selfing of single‐ramet plants was 44% less than multiple‐ramet plants, indicating that the contribution of between‐ramet geitonogamy was substantially less (20%) than within‐ramet geitonogamy, probably because of bumblebee foraging behavior. Our results demonstrate for the first time in a clonal plant that within‐ramet geitonogamy is substantially greater than between‐ramet geitonogamy and highlight the importance of considering the influence of clonal architecture and pollinator foraging on modes of self‐pollination.

## Introduction

Flowering plants possess diverse mating systems as a result of distinctive features of their biology, particularly their sedentary habit, hermaphroditic sex expression, and propensity for clonal growth. The predominance of hermaphroditism in angiosperms is associated with a wide range of mating patterns from predominant outcrossing to high levels of self‐fertilization, especially in self‐compatible species (Schemske and Lande 1985; Barrett and Eckert 1990; Goodwillie et al. 2005). The frequency of selfing is of considerable ecological and evolutionary significance because selfed offspring usually perform less well than those that are outcrossed, especially under field conditions (Darwin 1876; Charlesworth and Charlesworth 1987; Dudash 1990). However, the fitness consequences of selfing also require an understanding of the ecological factors affecting gene transmission through female and male function and this is determined, in part, by how, when, and how much self and outcross pollen is transported to stigmas by pollen vectors.

Understanding the diverse ecological and reproductive factors influencing selfing rates is a challenging problem in floral biology because self‐pollination can occur in contrasting ways resulting in different “modes of self‐fertilization” (Lloyd 1979; Barrett and Harder 1996). Modes of selfing are distinguished by the timing of self‐pollination, whether pollinators are involved, and whether self‐pollination occurs within or between flowers on a plant (Lloyd and Schoen 1992). Theoretical models indicate that how and when selfing occurs in relation to outcrossing has important consequences for parental fitness and mating system evolution (Lloyd 1979, 1992; Schoen et al. 1996). However, despite the impressive body of data on selfing rates in angiosperm plant populations, little is known about the modes of selfing in the vast majority of species.

A primary distinction in identifying modes of self‐fertilization is whether selfing occurs within (autogamy) or between flowers (geitonogamy) on a plant (Eckert 2000). These dissimilar modes of selfing have different costs and benefits and can therefore impose contrasting selective pressures on reproductive traits and patterns of mating. Self‐fertilization resulting from self‐pollination within a flower may be beneficial because it can ensure reproduction when outcross pollination is limited by a scarcity of pollinators and/or mates (reproductive assurance; reviewed in Eckert et al. 2006). Intrafloral selfing may also occur with little or no reduction in outcrossed seed production (seed discounting) or outcrossed siring success (pollen discounting), particularly if self‐pollination occurs autonomously and is delayed until after opportunities for outcrossing have passed (delayed selfing; Lloyd 1992; Harder and Wilson 1998). In animal‐pollinated plants, autogamous selfing may also be mediated by pollinator activities (facilitated and competing selfing; Lloyd 1992), and these modes of autogamous selfing are generally less advantageous.

Geitonogamous selfing involves the transfer of pollen between flowers in a manner analogous to outcrossing. It therefore requires the same floral investment and mechanisms employed for pollen dispersal between plants. As a result, geitonogamy provides little or no reproductive assurance and the ovules and pollen involved in selfing are prevented from participating in outcrossing. Thus, geitonogamy results in complete seed and pollen discounting (Lloyd 1992; de Jong et al. 1993; Harder and Barrett 1996; Harder and Wilson 1998). Because geitonogamy is almost never advantageous, it has most often been viewed as a nonadaptive byproduct of floral display (Lloyd 1992; Jarne and Charlesworth 1993; Harder and Barrett 1995; Eckert 2000). In animal‐pollinated species, the abundance and behavior of pollinators and their interaction with floral design and display are the primary determinants of the relative frequency of autogamous and geitonogamous selfing, although information directly linking pollination and mating is incomplete for most species.

Plant clonality has the potential to influence various aspects of mating in plant populations, particularly modes of selfing. If the expansion of genets by clonal growth is accompanied by large floral displays in self‐compatible species, local foraging by pollinators within a clone could increase selfing rates as a result of geitonogamy. This idea has been repeatedly emphasized in discussions of the potentially antagonistic interactions that can occur between clonal growth and sexual reproduction (Handel 1985; Charpentier 2002; Vallejo‐Marín et al. 2010; Barrett 2015). However, empirical evidence on the influence of clonal growth on mating patterns is mixed and appears to depend in large part on the size and clonal growth strategy of species (Reusch 2001; Routley et al. 2004; Albert et al. 2008; Liao et al. 2009; Matsuo et al. 2014; Van Drunen et al. 2015). Populations characterized by a high level of intermingling of ramets belonging to different genets are likely to experience less geitonogamy in comparison with populations composed of large intact clones that are spatially isolated from one another. Only a single study of clonal plants has investigated the contribution of different modes of selfing to the mating system. Implementing procedures involving floral emasculations and marker‐based estimates of selfing rate devised by Schoen and Lloyd (1992), Eckert demonstrated that in clonal *Decodon verticillatus,* 30% of the progeny resulted from selfing, and of these, 18% arose from autogamy, and 82% from geitonogamy (Eckert 2000). Through appropriate floral manipulations, he further decomposed the sources of geitonogamous selfing and revealed that they occurred about equally among three levels of clonal organization: within branches, between branches, and between ramets of a clone. Eckert concluded that the high rates of geitonogamy in *D. verticillatus* are unlikely to be adaptive and occurred because of the large plant size, mass flowering, and clonal growth that characterize this self‐compatible species (Eckert 2000).

Here, we experimentally investigated modes of selfing in the partially self‐compatible, bumblebee‐pollinated, clonal herb *Aconitum kusnezoffii* (Ranunculaceae). We also tracked the flowering phenology of clones to evaluate opportunities for different modes of self‐fertilization and investigated pollinator movements within and between ramets to assess patterns of pollen dispersal. We chose to investigate clonality and mating in *A*. *kusnezoffii* for two specific reasons. First, clones are easily distinguished in the field and only rarely grow intermingled. This facilitated the collection of data on flowering phenology, pollinator foraging, and the sampling of open‐pollinated seed families from different clones. Second, *A*. *kusnezoffii* is largely visited at our study sites by a single species of bumblebee, which is very abundant in populations and easily observed.

The primary objective of our study was to estimate the relative contributions of autogamy, within‐ramet geitonogamy, and between‐ramet geitonogamy to self‐fertilization in *A*. *kusnezoffii*. Our investigation had three main components: (1) We determined the contribution of autogamy to the total selfing rate of clones by comparing the amount of selfing in emasculated flowers with that experienced by intact flowers. (2) We estimated the amount of selfing caused by autogamy and within‐ramet geitonogamy by comparing the selfing rate of flowers on ramets in which all flowers on an inflorescence (raceme) were emasculated, to ramets from the same clone on which flowers were left intact. Because the emasculated flowers experience no autogamy or within‐ramet geitonogamy, only between‐ramet geitonogamy contributed to their selfing rates. (3) We measured the contribution of mating with genetically related clones (biparental inbreeding) by estimating the selfing rate of single‐ramet clones in which all flowers were emasculated. Because flowers on these ramets experienced no autogamy, within‐ramet geitonogamy, or between‐ramet geitonogamy, estimates of the selfing rate greater than zero could only arise from biparental inbreeding. The findings of our study provide new insights into mating patterns in clonal plants and enable an assessment of the consequences of multiple inflorescences within a clone for patterns of geitonogamous pollination.

## Materials and Methods

### Study species and sites

*Aconitum kusnezoffii* is a tall, perennial, clonal herb that is widespread in China and occurs on grassy slopes and forest margins, often by streams (Li and Kadota 2001). Populations are composed of clumps (clones) of flowering and nonflowering ramets produced from root tubers. Clones can be composed of between 1 and 28 flowering ramets and be up to 2 m in diameter. The hermaphroditic flowers are large, showy, blue, protandrous, and are produced in racemes with anthesis progressing acropetally (bottom‐to‐top). Flowers last for ~6 days with roughly 4 days in the male phase followed by 2 days in the female phase (Liao et al. 2009). Individual flowering ramets produce terminal racemes with between 2 and 34 flowers and daily display size averages 12 flowers. Ramets also produce a small number of lateral inflorescences, which commence flowering after terminal racemes have finished flowering, but in our study we excluded these from consideration. Experimental pollinations indicate that *A*. *kusnezoffii* is partially self‐compatible, with rates of pollen‐tube growth similar between self and outcross pollen with both reaching the ovary within 12 h. However, percent seed set following controlled self‐pollination (46%) is significantly lower than that from cross‐pollination (81%) as a result of early‐acting inbreeding depression and possibly a weak ovarian self‐incompatibility system (Liao et al. 2009; Hao et al. 2012).

At our study sites, plants are largely visited by *Bombus ignitus*, which is abundant during the flowering period (August–early September) and forages primarily for nectar. Other minor visitors are pollen‐collecting *Apis mellifera* and *Episyrphus balteatus,* which are ineffective as pollinators because they mostly fail to contact stigmas when visiting flowers (Liao et al. 2009).

We conducted pollinator observations and estimated modes of selfing in a population in Xiaolongmen National Forest Park, west Beijing, China (Population 1 in Liao et al. 2009; 39; °57′32.1″ N, 115°27′03.8″ E, 1034 m), and made records of flowering phenology in a second population (Population 4 in Liao et al. 2009, 39°58′05.5″ N, 115°25′48.0″E, 1188 m) that was 3 km from Population 1. A previous study of Population 1 in 2007 reported a selfing rate of 0.145 ± 0.016 (range 0.028 ± 0.000–0.204 ± 0.031), based on allozyme markers (Liao et al. 2009).

### Flowering phenology and pollinator observations

In 2008, we chose five clones in Population 4 and recorded the anthesis of all flowers on terminal racemes daily from 11 August to 5 September. We recorded if flowers were in their male or female phase based on whether at least one anther was dehiscent or stigmas were receptive. In Population 1, we observed numerous bumblebee visits to four clones on 23 August from 9:00 to 12:00 and 13:00 to 16:00 to investigate the potential influence of pollinator behavior on modes of selfing. We classified bee movements once they had arrived at a clone as either within‐ versus between‐ramet moves and within‐ramet moves as up or down the raceme.

### Floral manipulation experiment

Based on methods described in Eckert 2000, we used three floral manipulation treatments involving the removal of anthers from flowers. Flowers of *A*. *kusnezoffii* are easily emasculated, and this procedure does not alter pollinator attraction or damage female organs or nectaries (W. J. Liao unpublished). Treatments included single‐flower emasculation (SFE), whole‐ramet emasculation on multiple‐ramet clones (WEM), and whole‐ramet emasculation on single‐ramet clones (WES). For the SFE treatment, we randomly chose one flowering ramet from each of 50 clones distributed throughout Population 1 and marked the position of each flower on the terminal raceme from bottom‐to‐top at the beginning of flowering in 2007. We then removed anthers from flowers at even‐numbered positions before dehiscence and left flowers at odd‐numbered positions intact. On average, emasculation was applied to six flowers per ramet. For the WEM treatment, we randomly chose 50 multiple‐ramet clones distributed throughout the population and tagged two flowering ramets from each clone. On each of the clones, the flowers on the terminal raceme of one tagged ramet were all emasculated, and flowers on the other tagged ramet were left intact. On average, each terminal raceme of a ramet produced 10 flowers (range 6–17). For the WES treatment, we randomly chose 50 single‐ramet clones and divided them into two groups. One group was subjected to emasculation of all flowers on terminal racemes, whereas all flowers on terminal racemes of the second group were left intact. On average, each plant produced 9 flowers (range 6–15). Intact flowers in all three treatments were treated as a control group, which was used to evaluate the effect of emasculation in each treatment. At the end of the growing season in October, we collected fruits from all flowers in the experiment and stored them at 4°C until estimation of selfing rates. Due to herbivory and other forms of damage, fruits were collected from only 73 of the 150 clones in the experiment.

### Estimation of selfing rates in the experiment

To assess the selfing rate of the 73 clones, we sampled 5–10 seeds from all fruits collected from each clone resulting in an average of 72 seeds for each clone. Because seeds of *A*. *kusnezoffii* are moderately large, it was possible to conduct our analyses on seeds rather than seedlings, thus avoiding issues associated with differential germination of outcrossed and selfed seeds. The genotypes at polymorphic locus AAT (aspartate aminotransferase, EC 2.6.1.1), SKD‐1, SKD‐2 (shikimate dehydrogenase, EC 1.1.1.25), and PGD (6‐phosphogluconate dehydrogenase, EC 1.1.1.44) of the seed progeny were determined using the vertical slab polyacrylamide gel electrophoresis method described in detail in Liao et al. (2009).

We jointly estimated the selfing rate (*s*) of each treatment using the program MLTR (Ritland 2002). The expectation–maximization method was used to determine maximum‐likelihood estimates, and standard deviations were calculated based on 1000 bootstrap values using individual seed within families as the unit of resampling. We compared selfing rates for intact and emasculated treatments by comparing 1000 bootstrap values from their respective selfing rates. A significant difference between the two treatments was accepted if the distributions overlapped by less than 5%. The *P‐*value was determined based on the area of overlap between the distributions of selfing rates of the two treatments.

### Estimating modes of selfing

We estimated the proportion of seeds produced resulting from selfing (*s*) and outcrossing (*t*) for each experimental treatment using the allozyme data. The levels of intrafloral selfing (autogamy), within‐ramet, and between‐ramet geitonogamy were each estimated from the difference in *s* between intact and emasculated flowers using methods described in Schoen and Lloyd (1992). We applied the subscript 0 for intact flowers, 1 for single‐flower emasculations, and 2 for whole‐ramet emasculations on multiple‐ramet clones. Self‐fertilization includes components due to autogamy (*a*) and geitonogamy (*g*), which in our study is further divided into within‐ramet (*w*) and between‐ramet (*r*) geitonogamy so that: (*a *+ [*w *+* r*]) + *t *=* *1.

If a single flower is emasculated, the seed it produces after a pollinator visit must be the result of either cross‐pollination or geitonogamy, but not autogamy. For single‐flower emasculations, according to the model of Schoen and Lloyd (1992), *a*_0_ = *s*_0_−*g*_0,_ where $g_{0}=\frac{s_{1}\left(1-s_{0}\right)}{1-s_{1}}$, and *s*_0_ is the selfing rate for intact flowers in the WEM treatment.

The contribution of between‐ramet geitonogamous selfing (*r*_0_) to the total selfing rate is calculated directly from the selfing rate of flowers from whole‐ramet emasculation on multiple‐ramet clones. Because the selfing rate for intact flowers is *s*_*0*_ **= *a*_*0*_ *+ w*_*0*_ *+ r*_*0*_ and the rate for flowers from whole‐ramet emasculation on multiple‐ramet plants is *s*_2_ = *r*_2_, therefore:

$$r_{0}=\frac{s_{2}\left(1-s_{0}\right)}{1-s_{2}}$$

Accordingly, we calculated within‐ramet geitonogamy (*w*) as:

$$w_{0}=g_{0}-r_{0}=\frac{s_{1}\left(1-s_{0}\right)}{1-s_{1}}-\frac{s_{2}\left(1-s_{0}\right)}{1-s_{2}}$$

We obtained standard deviations for each component using the same calculations for estimates of selfing rate based on 1000 bootstraps, and a statistical departure from zero was accepted if less than 5% of the distribution of each selfing component was below zero.

## Results

### Flowering phenology and pollinator behavior

**Figure 1:**
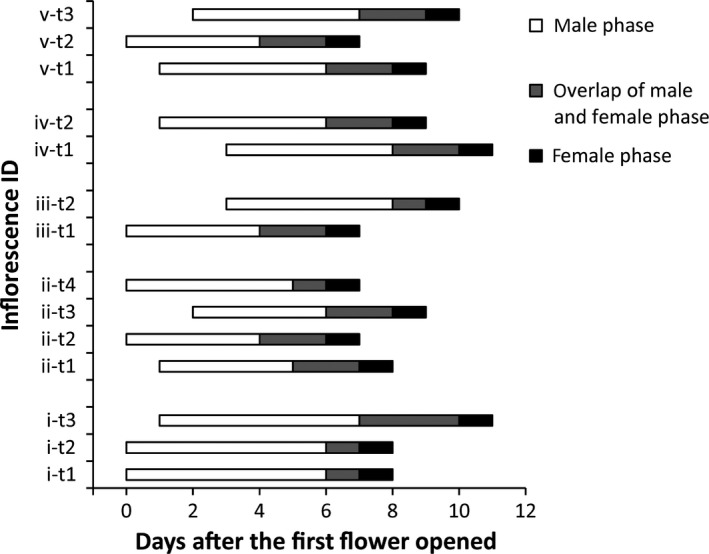
Flowering phenology of five clones of *Aconitum kusnezoffii* in the flowering season of 2008 in Population 4. Latin characters represent different clones. The *t* indicates a terminal raceme on a ramet. Arabic numerals represent different ramets. Each bar represents the period in which flowers in a terminal raceme are in male phase (white) followed by female phase (black). Overlap between flowers in different sex phases is indicated in gray.

Flowering of focal *A. kusnezoffii* clones in Population 4 began on 11 August and ceased on 5 September 2008. Each flowering ramet had a terminal raceme with 6–17 flowers. In the 14 ramets investigated from five clones, 48.1% of flowers had the opportunity to engage in within‐ramet geitonogamy. Flowering among ramets within a clone was near synchronized, and thus, flowers had the opportunity to be fertilized by pollen from other flowers on different ramets. As a whole, 62.6% of all flowers had the potential to be self‐fertilized via between‐ramet geitonogamy (Fig. 1).

We observed a total of 265 foraging bouts by individuals of *Bombus ignitus* visiting four clones of *A. kusnezoffii* during a single day. In 34 of these bouts, bees visited a single flower before departing from the clone. The remaining 231 foraging bouts involved visits to multiple flowers within a clone resulting in 1994 between‐flower pollinator movements. Within‐ramet movements accounted for 82% of the total movements we recorded, whereas between‐ramet movements within a clone accounted for 18% of visits. *Bombus ignitus* flew almost five times more often between flowers on a single ramet compared with between flowers on different ramets of a clone before departing. Approximately 64.6% of the within‐ramet movements were upward in direction and 35.4% were downward.

### Contributions of different modes of self‐fertilization

The emasculation of single flowers within racemes of *A. kusnezoffii* had a relatively small influence on levels of self‐fertilization (Table 1). The selfing rate of emasculated flowers (mean 0.131 ± SD. 0.022) was only 7% lower compared with that of intact flowers (0.141 ± 0.036), and this difference was not statistically significant (*P *=* *0.289), indicating autogamy contributes little to total selfing. The selfing rate of flowers subjected to whole‐ramet emasculation on multiple‐ramet clones (WEM) was 0.033 ± 0.002 compared to 0.149 ± 0.025 for unmanipulated flowers on multiple‐ramet clones. Thus, the WEM treatment reduced selfing by 78% (Table 1; *P *<* *0.05), indicating that within‐ramet geitonogamy plays an important role in contributing toward the total amount of self‐fertilization.

**Table 1:** Effect of floral manipulations on selfing rates (mean ± SD) in *Aconitum kusnezoffii*

Treatment	Selfing rate		*P‐*value
	Intact	Emasculated	
Single‐flower emasculation for multiple‐ramet plants	0.141 ± 0.036 (781/11)	0.131 ± 0.022 (776/11)	0.289
Whole‐ramet emasculation for multiple‐ramet plants	0.149 ± 0.025 (790/11)	0.033 ± 0.002 (711/10)	<0.05
Whole‐ramet emasculation for single‐ramet plants	0.083 ± 0.030 (1046/15)	0.002 ± 0.000 (1077/15)	<0.05

The numbers in parentheses following selfing rates are the number of progeny/number of maternal plants assayed for self‐fertilization. The *P* values were derived for comparisons between intact and emasculated flowers for each treatment.

The contribution of between‐ramet geitonogamy to the total selfing rate was obtained by comparing the selfing rate of clones with one flowering ramet (0.083 ± 0.030) to that of clones with multiple flowering ramets (0.149 ± 0.025). Because single‐ramet clones had no opportunity for between‐ramet geitonogamy, the selfing rate of single‐ramet clones was 44% less than that of multiple‐ramet clones (Table 1; *P *<* *0.05). This indicates that between‐ramet geitonogamy contributes significantly to the overall rates of selfing, albeit at a lower level than within‐ramet geitonogamy. Based on the model of Schoen and Lloyd (1992), autogamy was estimated to account for 12% of the selfing rate with the remaining 88% due to geitonogamy, which occurred mainly within flowering ramets of a clone (Table 2).

**Table 2:** Estimates of the components of selfing in *Aconitum kusnezoffii*

	*s*	*a*	*w*	*r*	*g*
Estimate:	0.149 ± 0.025	0.018 ± 0.008	0.102 ± 0.012	0.029 ± 0.011	0.131 ± 0.022
Proportion	100%	12%	68%	20%	88%
*P*	<0.001	0.067	<0.001	<0.001	<0.001

Total selfing (*s*), autogamous selfing (*a*), within‐ramet geitonogamy (*w*), between‐ramet geitonogamy (*r*), and total geitonogamy (*g*); (*s *= *a *+ *g*,* g *= *w *+ *r*). The standard deviation and probability that the estimate does not differ from zero were derived for each component based on 1000 bootstraps.

The contribution of biparental inbreeding to the mating system of *A. kusnezoffii* was estimated by examining the selfing rate of flowers on clones with one flowering ramet in which all flowers were emasculated. The emasculated flowers on these ramets had no opportunity for any mode of self‐fertilization. The selfing rate of emasculated flowers on single‐ramet plants (0.002 ± 0.000) was not significantly greater than zero (Table 1, *P* = 0.076). A minimum value of biparental inbreeding was also estimated based on the difference between the single‐ and multilocus selfing rates (Waller and Knight 1989). Subtraction of the single‐locus outcrossing rate from the multilocus outcrossing rate gave a minimum estimate of biparental inbreeding of −0.002 ± 0.002, further supporting that the estimate of biparental inbreeding for emasculated flowers on single‐ramet clones was close to zero.

## Discussion

Our floral manipulation experiment investigated mating patterns within and between ramets of clonal *A. kusnezoffii* using marker gene analysis. Two main findings emerged from our study: (1) The contribution of geitonogamy to the overall selfing rate of clones was 7 times greater than autogamy, and (2) within‐ramet and between‐ramet geitonogamy comprised 78% and 22% of the total amount of geitonogamy, respectively (Table 2). These patterns of mating can be explained, in part, by features of the floral biology of *A*. *kusnezoffii* and their interaction with its major pollinator—*Bombus ignitus*—the foraging behavior of which played the primary role in governing pollen dispersal within and between clones. We begin by considering the mechanisms responsible for the different components of selfing and the fitness costs associated with geitonogamy. We conclude by evaluating our results in light of recent studies that have questioned the widespread assumption that increased clone size is generally associated with elevated rates of between‐ramet geitonogamy.

### Mechanisms responsible for modes of self‐fertilization

We recorded relatively low (12%) rates of autogamous selfing (Table 2) providing quantitative support for the hypothesis that protandry should restrict opportunities for self‐pollination within a flower owing to the limited overlap between female and male function (Liao et al. 2009). Although flowers of *A*. *kusnezoffii* have protracted anther dehiscence over the 4 days of male function, high levels of visitation by *B. ignitus* rapidly depleted pollen from flowers, and thus, opportunities for pollinator‐mediated intrafloral self‐pollination were limited once stigmas became receptive. The main cause of self‐pollination in *A*. *kusnezoffii* was pollen transfer between flowers in different sex phases resulting in 88% geitonogamous selfing. This value is consistent with the few investigations that have used floral manipulations and marker genes to explicitly measure geitonogamy in self‐compatible, animal‐pollinated species. In annual *Impatiens pallida,* geitonogamous selfing was 10 times higher than autogamous selfing (Schoen and Lloyd 1992)*,* in *D. verticillatus,* 82% of total selfing resulted from geitonogamy (Eckert 2000), and in *Mimulus guttatus,* 50% of selfing resulted from autonomous selfing, with the remaining half evenly divided between geitonogamy and biparental inbreeding (Leclerc‐Potvin and Ritland 1994). Thus, geitonogamy is a major cause of selfing in animal‐pollinated plants that display several to many flowers simultaneously.

Our observations of flowering phenology and the foraging patterns of *B. ignitus* provide a functional explanation for the different modes of selfing revealed by our study. First, flowering within and between ramets of *A*. *kusnezoffii* clones was generally quite synchronous, although some degree of asynchrony was observed providing opportunities for between‐ramet geitonogamy. We estimated that 48.1% and 62.6% of the flowers we sampled had an opportunity to engage in within‐ramet versus between‐ramet geitonogamy, respectively. Moreover, of the nearly 2000 flower‐to‐flower movements by *B. ignitus* that we observed, individuals were 5 times more likely to move between nearby flowers on a single ramet than between more distant flowers on different ramets of a clone. These patterns were consistent with our finding that the majority of geitonogamous selfing resulted from within‐ramet geitonogamy (Table 2). Most (65%) bumblebee moves within inflorescences of *A*. *kusnezoffii* were upwards, confirming an earlier study of bumblebee flight patterns in this species (Ma et al. 2012). This negative geotactic pattern of foraging is commonly observed in the vertically orientated inflorescences of bee‐pollinated plants (Manning 1956; Pyke 1978; Barrett et al. 1994; Jordan and Harder 2006). In those like *A*. *kusnezoffii* with synchronized protandry, this foraging pattern should limit geitonogamous selfing and pollen discounting (Harder et al. 2000). However, a significant proportion (35%) of pollinator moves by *B. ignitus* were downward on inflorescences. These provide an opportunity for pollen transfer from male to female functioning flowers and explain the surprisingly high rates of intraramet geitonogamy revealed by our study.

One caveat is necessary when interpreting these data. Our estimates of modes of selfing and observations of pollinator foraging were obtained from a different population from which data on flowering phenology were made. However, other studies that we have conducted in populations of *A*. *kusnezoffii* including Population 1 (Liao et al. 2007, 2009; Ma et al. 2012) indicate that the flowering patterns that we observed in Population 4 are in no way exceptional. Also, although we only collected data on pollinator foraging from a single population, our observations of other populations indicate that *B. ignites* is the primary pollinator in this region and forages in an identical manner to what we observed in Population 1. We are therefore confident that the flowering patterns and pollinator foraging we report in this study are general and can explain the functional associations between flowering phenology, pollinator behavior, and patterns of mating in *A*. *kusnezoffii*.

### Limiting the costs of geitonogamy

Geitonogamy is the most costly mode of selfing because it results in complete pollen, ovule, and seed discounting and provides no reproductive assurance (Lloyd 1992). We have identified the proximal causes of geitonogamy experienced by clones of *A*. *kusnezoffii,* but determining empirically the precise fitness costs associated with geitonogamy is challenging and has not been attempted for any plant species, despite efforts at measuring gamete and seed discounting (Harder and Barrett 1995; Harder and Wilson 1998; Herlihy and Eckert 2002; Lau et al. 2008; Vaughton and Ramsey 2010; Busch and Delph 2012; Karron and Mitchell 2012). However, it is important to emphasize that despite our focus on modes of selfing in this study, the overall mating system of *A*. *kusnezoffii* is largely outcrossing, with an average of ~85% of offspring resulting from between‐clone mating. Moreover, we also estimated negligible levels of biparental inbreeding. High outcrossing rates in *A. kusnezoffii* are undoubtedly promoted by the reliable and abundant pollinator service provided by *B. ignitus,* despite the prevalence of within‐ramet foraging. Following the deposition of self and outcross pollen on stigmas, pollen‐tube growth prior to the entry of ovaries is similar between the two pollen types (Liao et al. 2009). Therefore, we can infer that reproductive processes operating in the ovary must play an important role in limiting geitonogamous selfing and maintaining high outcrossing rates in *A*. *kusnezoffii*.

The detrimental effects of geitonogamy on progeny fitness in *A*. *kusnezoffii* are diminished by two separate postpollination mechanisms (Hao et al. 2012). A partial prezygotic late‐acting self‐incompatibility appears to limit the frequency of ovule fertilization by self‐pollen tubes but without preventing selfing altogether (Hao et al. 2012). More importantly, significant amounts of self‐fertilized embryos are aborted during seed maturation due to early‐acting inbreeding depression. Predispersal inbreeding depression in *A*. *kusnezoffii* has been estimated to be 0.502, based on the analysis of fruit and seed set following controlled self‐ and cross‐pollinations (Liao et al. 2009). Thus, weak late‐acting self‐incompatibility serves to reduce rates of self‐fertilization and early‐acting inbreeding depression terminates the development of large numbers of selfed embryos. The abortion of selfed seeds before they are fully mature prevents significant amounts of maternal resources from being allocated to progeny. Saved resources are probably used for future clonal growth and reproduction.

Geitonogamy is ubiquitous in self‐compatible flowering plants with large floral displays. Indeed, it has been proposed that the mating costs associated with geitonogamy act as an important selective force shaping the evolution of floral design and display (Harder and Barrett 1996), particularly in clonal species (Vallejo‐Marín et al. 2010). However, the extent to which increased clone size results in greater levels of geitonogamous selfing and reduced fitness has recently been challenged by studies showing that clonality under some circumstances may increase the quantity and quality of mating (Liao and Harder 2014; Van Drunen et al. 2015). These studies point to the importance of considering clonal architecture and the nature of pollen dispersal both within and between clones in evaluating the extent to which clonality influences mating patterns and fitness. Somewhat unexpectedly our study has demonstrated that most geitonogamy occurs within rather than between ramets of a clone. This suggests that in species like *A*. *kusnezoffii,* clonal expansion may not necessarily be accompanied by large increases in between‐ramet geitonogamy. Future research on modes of selfing in flowering plants with contrasting clonal strategies is warranted to determine the intensity of geitonogamy and how it occurs.
